# Clinical Characteristics and Long-Term Prognosis of Elderly Valvular Heart Disease Patients with Diabetes Mellitus: Five-Year Experience from a Single-Center Study of Southern China

**DOI:** 10.1155/2021/2558639

**Published:** 2021-10-27

**Authors:** Yuan-Feng Liang, Feier Song, Huixia Liu, Jian Liu, Yu-Yuan Zhang, Wei-Dong Lin, Hong-Tao Liao, Hui-Ming Guo, Gary Tse, Fang-Zhou Liu, Zhanyi Lin

**Affiliations:** ^1^Department of Geriatrics, Guangdong Provincial Geriatrics Institute, Guangdong Provincial People's Hospital, Guangdong Academy of Medical Sciences, Guangzhou 510080, China; ^2^Department of Emergency and Critical Care Medicine, Guangdong Provincial People's Hospital, Guangdong Academy of Medical Sciences, Guangzhou 510080, China; ^3^Department of Cardiac Surgery, Guangdong Provincial Cardiovascular Institute, Guangdong Provincial People's Hospital, Guangdong Academy of Medical Sciences, Guangzhou 510080, China; ^4^Department of Cardiology, Atrial Fibrillation Center, Guangdong Provincial Cardiovascular Institute, Guangdong Provincial People's Hospital, Guangdong Academy of Medical Sciences, Guangzhou 510080, China; ^5^Department of Medicine and Therapeutics, Faculty of Medicine, Chinese University of Hong Kong, Shatin, Hong Kong, China; ^6^Li Ka Shing Institute of Health Sciences, Faculty of Medicine, Chinese University of Hong Kong, Shatin, Hong Kong, China; ^7^Smidt Heart Institute, Cedars-Sinai Medical Center, Los Angeles 90048, CA, USA; ^8^Department of Cardiology, Guangdong Provincial Cardiovascular Institute, Guangdong Provincial People's Hospital, Guangdong Academy of Medical Sciences, Guangzhou 510080, China

## Abstract

**Background:**

Diabetes mellitus (DM) is a prognostic marker in elderly patients with cardiovascular diseases, but its predictive value in elderly valvular heart disease (VHD) patients is unclear. This study aimed to investigate the effect of DM on the long-term outcome of elderly VHD patients.

**Methods:**

This single-center, observational study enrolled patients aged 65 and older consecutively with confirmed VHD using echocardiography. Patients, divided into the DM group and non-DM group, were followed up for major adverse cardiac and cerebrovascular events (MACCEs), including all-cause death, ischemic stroke, and heart failure rehospitalization.

**Results:**

Our study consisted of 532 patients over a median follow-up of 52.9 months. Compared with the non-DM group (*n* = 377), the DM group (*n* = 155) had higher incidences of ischemic stroke (25.2% vs. 13.5%, *P*=0.001), heart failure rehospitalization (37.4% vs. 20.7%, *P* < 0.001), and MACCEs (60.0% vs. 35.8%, *P* < 0.001). After adjustment of confounders by the multivariable cox regression, DM appeared as an independent predictor for MACCEs (adjusted hazard ratio, aHR: 1.88; 95% confidence interval 1.42–2.48; *P* < 0.001). In the subgroup analysis of VHD etiology and functional style, conversely, DM was a protective factor for MACCEs in the patients with rheumatic VHD compared with those without rheumatic VHD (aHR: 0.43 vs. 2.27, *P*=0.004).

**Conclusions:**

DM was an independent predictor for ischemic stroke and heart failure rehospitalization in elderly VHD patients undergoing conservative treatment.

## 1. Introduction

Valvular heart disease (VHD) has become a common cardiovascular condition in clinical practice. The prevalence of VHD is increasing over the age and ranges from 4 to 9% for those aged 65–75 years and ranges from 12 to 13% for those aged >75 years in the United States [1]. Besides, elderly VHD patients are not only highly associated with cardiac dysfunction and cardiac mortality but also aggravate the socioeconomic burden [2]. Valve replacement therapies are the treatment of choice when severe valvular damage occurs [3], but valve repair or replacement surgery may be associated with an increased risk of morbidity and mortality in elderly VHD patients [4, 5]. The short-term mortality rates of valve surgery in patients aged over 80 years were about 8–20% [6, 7]. Little clinical research has focused on elderly VHD patients receiving conservative treatment.

About 463 million people have been suffering from diabetes mellitus (DM) all over the world, with a significantly shortened life expectancy. The prevalence of DM is 9.3% in the world, and the number of patients will probably rise to 578 million by 2030 [8]. The degeneration of the cardiac tissue, especially the cardiac valve, is possibly prompted by impaired insulin sensitivity and uncontrolled hyperglycemia [9]. The relationship between DM and long-term cardiac and cerebrovascular disease outcomes in elderly VHD patients remains unclear. The present study aimed to investigate the effect of DM on the long-term clinical outcomes in elderly VHD patients receiving conservative treatment.

## 2. Methods

### 2.1. Study Design

The present study was a single-center, retrospective, observational study and reviewed all patients aged 65 years and older with VHD who were consecutively referred to the Department of Cardiology, Guangdong Provincial People's Hospital from January 1, 2010, to December 31, 2010. The exclusion criteria were as follows: (a) a previous history of valvular surgery or undergoing surgery during the follow-up period; (b) current malignant diseases or life expectancy of less than 12 months; and (c) refusal to follow-up. The patients were divided into the DM and non-DM groups regarding their history of DM. Demographic characteristics, concomitant diseases, and ongoing pharmacological treatment of all patients were retrieved from the electrical medical records, such as atrial fibrillation (AF), hypertension, DM, chronic kidney disease (CKD), and chronic obstructive pulmonary disease (COPD). By monitoring the comorbid conditions, as well as the patients' preferences, the therapeutic strategy was carried out based on the progression of the valvular defect and at the discretion of the cardiac multidisciplinary team. This study was approved by the Institutional Review Board at Guangdong Provincial People's Hospital, Guangzhou, China, and written informed consent was unnecessary due to the nature of the retrospective design.

### 2.2. Definitions

Diseases diagnosed were based on the 10th version of the International Classification of Diseases definitions from the World Health Organization. DM was defined as fasting plasma glucose ≥7.0 mmol/l (126 mg/dl) or 2 h plasma glucose ≥11.1 mmol/l (200 mg/dl) [10]. Notably, hypoglycemic treatments for patients with diabetes were based on the clinical evaluation of the disease and at the discretion of the physicians, and the level of blood glucose was well controlled.

All patients were examined by transthoracic echocardiography or transesophageal echocardiography. Left atrial diameter (LAd), left ventricular end-diastolic (LVDd), end-systolic diameter (LVSd), and left ventricular ejection fraction (LVEF) were measured according to the recommendations of the American Society of Echocardiography [11].

The valvular function was recorded as the following four styles: mitral stenosis (MS), mitral regurgitation (MR), aortic stenosis (AS), and aortic regurgitation (AR), according to the international recommendations and guidelines [12, 13]. Ischemic VHD was diagnosed by a medical history of ischemic heart disease, including the mitral regurgitation caused by the ventricular septal defect and the ischemic papillary muscle dysfunction after myocardial infarction [14, 15]. Infective VHD was defined according to the proposed modifications to the Duke criteria [16], including leaflet perforation or chordal rupture from infective endocarditis. Degenerative VHD was identified by the echocardiographic criteria [17] for senile mitral valve prolapse and calcific valvular lesions. Rheumatic VHD was defined based on a medical history of rheumatic fever and/or rheumatic valve changes [18, 19] with commissural fusion or leaflet restriction, especially an opening snap following the second heart sound and subsequent apical diastolic murmur. Congenital VHD was recognized as a congenital defect of the valve(s) by echocardiography with or without contrast imaging and myocardial strain imaging [20]. The underlying etiology of VHD was determined based on the corresponding medical history and echocardiographic presence.

### 2.3. Endpoints

The primary endpoint was defined as the major adverse cardiac and cerebrovascular events (MACCEs), including all-cause mortality, ischemic stroke, and heart failure rehospitalization. The secondary endpoints were defined as (1) all-cause mortality, (2) ischemic stroke, and (3) heart failure rehospitalization. Follow-up was performed by telephone interviews and out-patient visits at 6 months, 1 year, 2 years, 4 years, and anytime patients suffered from an endpoint event.

### 2.4. Statistical Analysis

Patients were divided into the DM group and non-DM group to examine the baseline characteristics and the primary and secondary endpoints. Categorical variables were presented as frequencies (percentages) and compared by the chi-square or Fisher's exact test. Continuous variables were expressed as mean ± standard deviation or median (interquartile range, IQR) and compared by Student's *t*-test or Wilcoxon test, depending on the distribution of data. The event-free survival was analyzed using the Kaplan–Meier method. The assessment period began from the time of cohort inception to the date of diagnosis of the endpoint event or end of follow-up (October 30, 2014), whichever came first. The cox proportional hazard model was built to identify independent predictors for the primary and secondary endpoints. The adjusted hazard ratio (aHR) and 95% confidence interval (CI) were calculated, by adjusting for the independent risk factors of MACCEs, which included sex, age ≥ 75 years, angiotensin-converting enzyme inhibitors/angiotensin receptor blockers, antithrombotic drugs, COPD, CKD, hypertension, AF, DM, LVEF < 50%, AR, AS, MR, and MS. The interaction between diabetes and other risk factors was tested using the Wald test. All reported *P* values were 2-sided, and statistical significance was set at *P* < 0.05. Statistical analysis was performed using R software (version 3.6.1; R Core Team, Vienna, Austria) and GraphPad Prism 8.0 (GraphPad Software, San Diego, California, USA).

## 3. Results

A total of 612 patients were reviewed, excluding 31 patients with a previous history of surgery during follow-up, 36 patients with a malignant tumor, and 13 patients lost to follow-up. Finally, 532 patients were included in the present study (median age of 75.0 (IQR: 70.0–80.0) years; 291 males). The first two places in the VHD etiology were degeneration (38.7%) and ischemia (28.4%), respectively. There were 155 patients in the DM group and 377 in the non-DM group. Compared with the non-DM group, there was no significant difference in baseline characteristics in the DM group, except a higher hypertension prevalence (81.9% vs. 61.3%, *P* < 0.001) and a larger LAd (41.9 (IQR: 37.0–47.5) vs. 40.0 (IQR: 35.0–45.0) mm, *P*=0.021) (Table 1).

**Figure 1:**
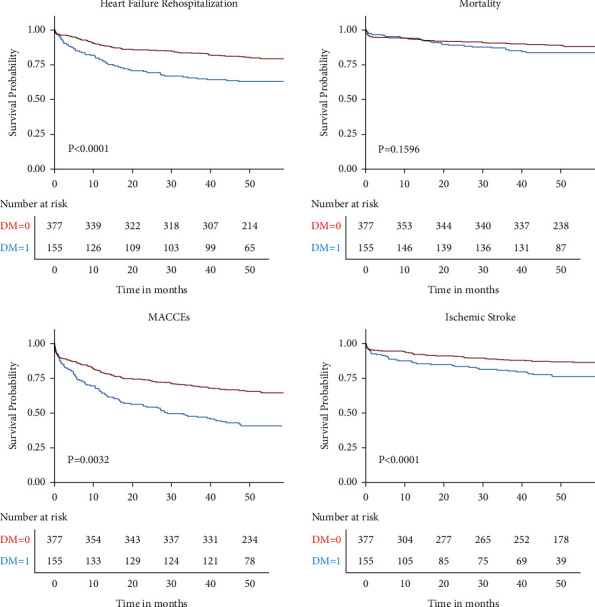
Kaplan–Meier event-free survival curves for composite endpoints (all-cause mortality, ischemic stroke, and heart failure rehospitalization) in elderly VHD patients with diabetes vs. nondiabetes. MACCEs, major adverse cardiac and cerebrovascular events; VHD, valvular heart disease.

Over a median follow-up of 52.9 (range: 46.6 to 58.8) months, 228 (42.9%) patients developed MACCEs, including 90 (16.9%) patients with ischemic stroke, 136 (25.6%) with heart failure rehospitalization, and 71 (13.4%) deaths. The DM group had higher incidences of ischemic stroke (25.2% vs. 13.5%, *P*=0.001), heart failure rehospitalization (37.4% vs. 20.7%, *P*=0.001), and MACCEs (60.0% vs. 35.8%, *P* < 0.001) than those in the non-DM group. However, there was no significant difference in all-cause mortality observed between these two groups (16.1% vs. 12.2%, *P*=0.226) (Table 2). The Kaplan–Meier event-free survival curves showed better long-term survival and prognosis in the non-DM group (Figure 1).

Under the analysis of multivariable cox regression, DM (aHR: 1.88; 95% CI: 1.42 to 2.48), age (aHR: 1.03; 95% CI: 1.01 to 1.06), hypertension (aHR: 1.60; 95% CI: 1.16 to 2.21), atrial fibrillation (aHR: 1.37; 95% CI: 1.03 to 1.84), and use of beta-blocker (aHR: 2.26; 95% CI: 1.51 to 3.38) were independent predictors for MACCEs after adjustment (Table 3). The relationship of cardiac risk factors to all-cause mortality is estimated in Table S1. Age and New York Heart Association functional class were independent predictors for all-cause mortality after adjustment. DM was an independent predictor of ischemic stroke (Table S2) and heart failure rehospitalization (Table S3).

**Figure 2:**
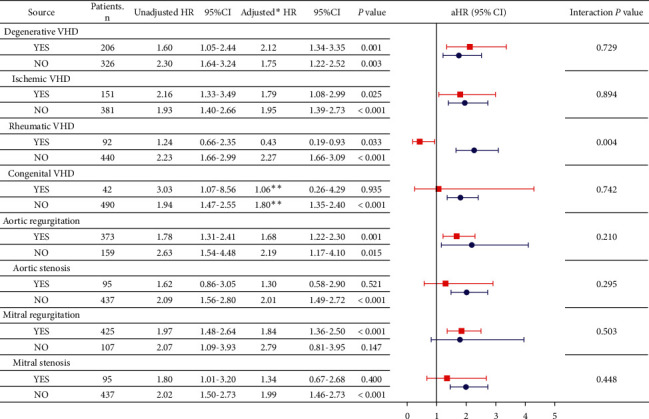
Primary outcome in subgroups of elderly VHD patients. VHD, valvular heart disease; HR, hazard ratio; CI, confidence interval. ^*∗*^Adjusted for age, chronic obstructive pulmonary disease, chronic kidney disease, hypertension, atrial fibrillation, New York Heart Association class, left atrial diameter, aortic regurgitation, beta-blocker, and diuretic. ^*∗∗*^Adjusted for age, chronic obstructive pulmonary disease, chronic kidney disease, hypertension, atrial fibrillation, New York Heart Association class, left atrial diameter, aortic regurgitation, and diuretic.

A further subgroup analysis was performed to assess the association between DM and MACCEs in various subgroups of VHD etiology and functional style (Figure 2). Compared with the subgroup of nonrheumatic VHD, a protective effect of DM for MACCEs was observed in the subgroup of rheumatic VHD (aHR: 0.43 vs. 2.27, *P*=0.004). Conversely, DM did not show a significant interaction effect for MACCEs in other VHD etiologies or in each functional style.

## 4. Discussion

### 4.1. Main Findings

To our best knowledge, this is the first study to directly determine the influence of DM on the clinical characteristics and the long-term outcome in elderly VHD patients. The main findings were as follows: (1) there were higher incidences of MACCEs, ischemic stroke, and heart failure rehospitalization observed in elderly VHD patients with DM; (2) DM was an independent hazardous factor for MACCEs in elderly VHD patients; (3) there was an underlying protective effect of DM on MACCEs observed in the subgroup of rheumatic VHD.

### 4.2. Etiology and Comorbidities of VHD Patients

Globally, VHD is commonly degenerative and mainly affects elderly patients with multiple comorbidities [19]. There is little literature focusing on the prognostic value of risk factors in elderly VHD patients. In the present study, the clinical characteristics and management of patients aged 65 and older were examined between the DM group and the non-DM group. The findings might be considered as a real-world picture of current VHD in elderly patients in Southern China.

With the improvement of the quality of life and medical conditions in China, the prevalence of degenerative VHD increased while rheumatic VHD decreased. In the present study, degenerative VHD was found in 38.7% of elderly patients, followed by ischemic VHD (28.4%) and rheumatic VHD (17.3%), because of the better socioeconomic status and living conditions in Guangdong province compared to the rest of China. In addition, compared with the non-DM group, the DM group had a higher hypertension prevalence (81.9% vs. 61.3%) and a larger LAd (41.9 vs. 40.0 mm). According to previous studies, 70–80% of patients with DM have hypertension [21]. A larger LAd in the DM group highlighted the alteration in atrial architecture and function as an underlying foundation of heart failure.

### 4.3. The Prognosis of VHD

The valve lesions impeded normal blood flow and increased the burden on the heart in the absence of symptoms at the beginning of the VHD course. Due to the slow and insidious progression of lesions, many patients might neglect the symptoms which gradually limit their activity level over the years. Once valvular stenosis or insufficiency occurred, it would induce damage to the heart, lead to abnormal heart function and heart failure, or even cause sudden death, because the unpredictable adverse consequences of severe VHD primarily affected the status of the ventricles and pulmonary circulation.

As for the elderly VHD patients, the potential benefits of valve surgery reduced while the risks increased, because they suffered from frailty, impaired cerebral perfusion, or complicated structural anatomy, including atherosclerosis in the aorta and mitral annular calcification [4, 5], with higher mortalities for the mitral valve, multiple valves, and concomitant coronary artery bypass grafting surgery [6, 7]. In the patients undergoing surgical aortic valve replacement, the octogenarians had higher morbidity and mortality, ranging from 5% to 18%, than the younger patients [22–25]. The increasing comorbidities with age and the high prevalence of postoperative atrial fibrillation in elderly patients undergoing valve replacement surgery were associated with significant morbidity and mortality [26, 27]. Survival was not the priority when considering therapeutic strategy in many elderly VHD patients. Only 7% of the elderly patients undergoing transcatheter aortic valve replacement cited improved survival as the reason for seeking treatment, but maintaining independence (30%) and the ability to do a specific activity (48%) were the predominant reasons [28]. The main goals of treatment in elderly VHD patients, including quality of life, functionality, independence, and palliation of severe symptoms, took precedence over meaninglessly increased longevity [29].

VHD in elderly patients, associated with an alarming risk of mortality, heart failure, and stroke, is a highly morbid condition [30]. In the present study, despite the similar all-cause mortality between the two groups (16.1% in the DM group and 12.2% in the non-DM group), it is worth noting that elderly VHD patients with DM suffered a higher risk of ischemic stroke and heart failure rehospitalization. Decompensated heart failure was the main reason for admission of VHD patients, reflecting the long-term evolution of VHD and the severity of the clinical condition [31].

We identified important predictors for MACCEs in elderly VHD patients and confirmed the different components' predictive power in the subgroups. Future studies are needed to further investigate their impact on prognosis in the specific population. Nevertheless, optimal management of these risk factors was warranted, given the other established cardiovascular benefits [32].

### 4.4. Impact of DM

In general, DM, associated with a 2.5-fold increased risk of ischemic stroke [33], was an established risk factor for ischemic strokes, including lacunar, large artery, and cardioembolic stroke [34]. The present study showed that DM held a 1.74-fold increased risk of ischemic stroke in elderly VHD patients undergoing conservative treatment. The potential pathogenesis is complex, multifactorial, and incompletely understood. The microvascular disease had been shown to affect many organs, including the brain, in DM patients [35]. It was reported to be a potential mechanism associated with an increased risk of lacunar ischemic stroke [36].

Major causes of heart failure in patients with DM were aging, coronary artery disease, CKD, and hypertension [37, 38]. Similarly, in the present study, DM was identified as an independent predictor for heart failure rehospitalization in elderly VHD patients, compared with the non-DM group (37.4% vs. 20.7%, respectively). Given the aging population and growing prevalence of VHD, the latent pathogenesis explaining diabetic complications in myocardial dysfunction may be the direct effect of insulin resistance or hyperglycemia on the myocardium [39].

Previous studies had proposed numerous pathophysiological or molecular mechanisms, including increased polyol and hexosamine pathways, oxidative stress, and activation of the diacylglycerol/protein kinase C pathway. Moreover, alterations in signal transduction pathways induced by hyperglycemia or toxic metabolites could also lead to vascular and cellular dysfunctions, such as abnormal hemodynamics and increased apoptosis by altering genetic expression and protein function [40].

DM was reported in 34% of the elderly patients undergoing valve replacement for severe AS [41]. There was a higher risk for postoperative sternal wound infections, respiratory failure, renal failure, blood transfusions, and in-hospital mortality in diabetic patients than in nondiabetic patients because of the multisystem inflammation and dysfunction in diabetes [42, 43]. However, few studies focused on elderly VHD patients receiving conservative treatment. In our study, the long-term mortality was similar in elderly VHD patients with or without DM. The conservative option might explain the result since it avoided postoperative complications and might reduce multisystem inflammation.

### 4.5. Subgroup Analysis

In the present study, compared with the subgroup of nonrheumatic VHD, a protective effect of DM for MACCEs was observed in the subgroup of rheumatic VHD (aHR: 0.43 vs. 2.27, *P*=0.004). The most likely explanation is the use of hypoglycemic agents against inflammatory immune responses. The inflammation of recurrent rheumatic fever is associated with a worsening of rheumatic heart disease. Inflammatory immune cells are activated from a low-energy-consumption resting state to a high-metabolism active state by greatly increased glucose consumption [44]. Glucose transporter1 (GLUT1) is upregulated in activated inflammatory cells, in concert with an extreme increase in glucose consumption, contributing to proinflammatory cellular responses. At the same time, GLUT1 and its family make the surrounding tissue insulin-resistant and the remaining glucose is increased to supply proinflammatory cellular responses [45].

In detail, metformin notably downregulates the expression of GLUT1 and reduces the secretion of proinflammatory mediators IL-6, IL-8, and MCP-1 by increasing the phosphorylation of adenine monophosphate-activated protein kinase. Furthermore, metformin promotes inflammatory resolution by altering cellular metabolic activity [46]. Apart from metformin, peroxisome proliferator-activated receptor *γ* agonists reduce disease activity and incidence of rheumatoid arthritis [47]. Moreover, dipeptidyl peptidase-4 inhibitors in type 2 diabetes may reduce the risk of autoimmune diseases [48]. Most of the hypoglycemic agents treat hyperglycemia through altering glucose metabolism and alleviating insulin resistance, which may reduce the activation of inflammatory immune cells, thereby relieving inflammation and immune response, including rheumatic heart disease. However, the clinical relationship between hypoglycemic agents and rheumatic heart disease is still unclear, which requires further studies to estimate.

### 4.6. Limitations

Firstly, owing to the nature of the observational study, it was not able to infer the direct causal effect of DM on the long-term prognosis. Secondly, echocardiographic findings might vary due to diagnostic modalities and sonologists' practice. Thirdly, as the enrollment started in 2010, glycated hemoglobin A1c was not included either in the diagnostic criteria of DM in 2010 as previously mentioned or for the prognostic value in the cox regression. Despite these limitations, the present study, which was unique in its size and in being the first study to explore the longitudinal outcomes in elderly VHD patients, added valuable new information regarding comorbid diabetes and offered a long-term follow-up. Large-scale prospective studies specific to elderly diabetic adults are needed to provide more evidence.

## 5. Conclusion

In conclusion, VHD patients aged 65 and older with DM were at high risk of ischemic stroke, heart failure rehospitalization, and MACCEs. DM was an independent predictor for MACCEs in elderly VHD patients undergoing conservative treatment. However, the presence of rheumatic VHD as a significant modifier for the predictive value of DM for MACCEs needs further research.

## Figures and Tables

**Table 1:** Baseline characteristics stratified by presence of diabetes mellitus in elderly VHD patients.

	Overall (*n* = 532)	Diabetes (*n* = 155)	Nondiabetes (*n* = 377)	*P* value
Age (years)	75.0 (70.0–80.0)	75.0 (71.5–79.0)	75.0 (70.0–80.0)	0.556
Male, *n* (%)	291 (54.7%)	90 (58.1%)	201 (53.3%)	0.317
Medical history				
COPD, *n* (%)	116 (21.8%)	30 (19.4%)	86 (22.8%)	0.380
Atrial fibrillation, *n* (%)	232 (43.6%)	77 (49.7%)	155 (41.1%)	0.070
Hypertension, *n* (%)	358 (67.3%)	127 (81.9%)	231 (61.3%)	<0.001
Chronic kidney disease, *n* (%)	66 (12.4%)	25 (16.1%)	41 (10.9%)	0.095
Congenital VHD, *n* (%)	42 (7.9%)	9 (5.8%)	33 (8.8%)	0.252
Ischemic VHD, *n* (%)	151 (28.4%)	50 (32.3%)	101 (26.8%)	0.204
Infective VHD, *n* (%)	7 (1.3%)	2 (1.3%)	5 (1.3%)	>0.999
Degenerative VHD, *n* (%)	206 (38.7%)	62 (40.0%)	144 (38.2%)	0.698
Rheumatic VHD, *n* (%)	92 (17.3%)	26 (16.8%)	66 (17.5%)	0.839
NYHA class, *n* (%)				0.726
I	30 (5.6%)	8 (5.2%)	22 (5.8%)	
II	274 (51.5%)	86 (55.5%)	188 (49.9%)	
III	184 (34.6%)	49 (31.6%)	135 (35.8%)	
IV	37 (7.0%)	11 (7.1%)	26 (6.9%)	
NYHA class > 2, *n* (%)	221 (41.5%)	60 (38.7%)	161 (42.7%)	0.349
Echocardiographic findings				
LAd (mm)	40.0 (35.0–46.0)	41.9 (37.0–47.5)	40.0 (35.0–45.0)	0.021
LVDd (mm)	47.5 (43.0–54.3)	47.0 (42.0–52.0)	48.0 (43.0–56.0)	0.168
LVSd (mm)	29.0 (25.0–36.0)	28.0 (25.0–33.0)	30.0 (25.0–37.0)	0.123
LVEF (%)	63.5 (55.0–68.0)	64.0 (57.5–68.0)	63.0 (53.0–69.0)	0.522
LVEF < 50%, *n* (%)	101 (19.0%)	25 (16.1%)	76 (20.2%)	0.522
Mitral regurgitation, *n* (%)				0.07195
None	107 (20.1%)	28 (18.1%)	79 (21.0%)	
Mild	175 (32.9%)	53 (34.2%)	122 (32.4%)	
Moderate	92 (17.3%)	36 (23.2%)	56 (14.9%)	
Severe	158 (29.7%)	38 (24.5%)	120 (31.8%)	
Mitral stenosis, *n* (%)				0.2481
None	437 (82.1%)	120 (77.4%)	317 (84.1%)	
Mild	35 (6.6%)	11 (7.1%)	24 (6.4%)	
Moderate	27 (5.1%)	11 (7.1%)	16 (4.2%)	
Severe	33 (6.2%)	13 (8.4%)	20 (5.3%)	
Aortic regurgitation, *n* (%)				0.1219
None	159 (29.9%)	39 (25.2%)	120 (31.8%)	
Mild	233 (43.8%)	77 (49.7%)	156 (41.4%)	
Moderate	90 (16.9%)	29 (18.7%)	61 (16.2%)	
Severe	50 (9.4%)	10 (6.5%)	40 (10.6%)	
Aortic stenosis, *n* (%)				0.8668
None	437 (82.1%)	125 (80.6%)	312 (82.8%)	
Mild	55 (10.3%)	16 (10.3%)	39 (10.3%)	
Moderate	17 (3.2%)	6 (3.9%)	11 (2.9%)	
Severe	23 (4.3%)	8 (5.2%)	15 (4.0%)	
Medication				
*β*-Blockers, *n* (%)	367 (69.0%)	108 (69.7%)	259 (68.7%)	0.825
ACEI/ARB, *n* (%)	163 (30.6%)	55 (35.5%)	108 (28.7%)	0.120
Antithrombotic drugs, *n* (%)	234 (44.0%)	78 (50.3%)	156 (41.4%)	0.059
Diuretic agents, *n* (%)	109 (20.5%)	29 (18.7%)	80 (21.2%)	0.515

*Note*. Data were expressed as *n* (%) or median (interquartile range). COPD, chronic obstructive pulmonary disease; ACEI, angiotensin-converting enzyme inhibitors; ARB, angiotensin receptor blockers; NYHA, New York Heart Association; LAd, left atrium diameter; LVDd, left ventricular internal diameter at end-diastole; LVSd, left ventricular internal diameter at end-systole; LVEF, left ventricular ejection fraction; and VHD, valvular heart disease.

**Table 2:** Outcomes according to diabetes mellitus in elderly VHD patients.

	Total (*n* = 532)	Diabetes (*n* = 155)	Nondiabetes (*n* = 377)	*P* value
Ischemic stroke	90 (16.9%)	39 (25.2%)	51 (13.5%)	0.001
Heart failure rehospitalization	136 (25.6%)	58 (37.4%)	78 (20.7%)	<0.001
All-cause mortality	71 (13.4%)	25 (16.1%)	46 (12.2%)	0.226
MACCEs	228 (42.9%)	93 (60.0%)	135 (35.8%)	<0.001

*Note*. Data are presented as *n* (%). MACCEs, major adverse cardiac and cerebrovascular events; VHD, valvular heart disease.

**Table 3:** Multivariable cox regression analysis for MACCEs in elderly VHD patients.

	aHR	95% CI	*P* value
Age	1.03	1.01–1.06	0.002
COPD	1.32	0.97–1.80	0.076
Chronic kidney disease	1.21	0.85–1.72	0.300
Hypertension	1.60	1.16–2.21	0.004
Atrial fibrillation	1.37	1.03–1.84	0.033
Diabetes mellitus	1.88	1.42–2.48	<0.001
NYHA class	1.16	0.93–1.44	0.192
LAd	1.00	0.98–1.02	0.985
Aortic regurgitation	1.25	0.92–1.70	0.154
Beta-blocker	2.26	1.51–3.38	<0.001
Diuretic	1.16	0.84–1.60	0.373

*Note*. MACCEs, major adverse cardiac and cerebrovascular events; VHD, valvular heart disease; COPD, chronic obstructive pulmonary disease; NYHA, New York Heart Association; LAd, left atrial diameter; aHR, adjusted hazard ratio; and CI, confidence interval.

## Data Availability

All data generated or analyzed during this study are included in this published article.

## Supplementary Materials

Supplementary Materials Table S1: multivariable cox regression analyses for all-cause mortality in elderly VHD patients. Table S2: multivariable cox regression analyses for ischemic stroke in elderly VHD patients. Table S3: multivariable cox regression analyses for heart failure rehospitalization in elderly VHD patients.

## References

[1] Nishimura R. A., Otto C. M., Bonow R. O. (2014). 2014 AHA/ACC guideline for the management of patients with valvular heart disease: a report of the American College of Cardiology/American Heart Association task force on practice guidelines. *The Journal of Thoracic and Cardiovascular Surgery*.

[2] D’Arcy J. L., Coffey S., Loudon M. A. (2017). Large-scale community echocardiographic screening reveals a major burden of undiagnosed valvular heart disease in older people: the OxVALVE population cohort study. *European Heart Journal*.

[3] Nishimura R. A., Otto C. M., Bonow R. O. (2017). 2017 AHA/ACC focused update of the 2014 AHA/ACC guideline for the management of patients with valvular heart disease: a report of the American College of Cardiology/American Heart Association task force on clinical practice guidelines. *Circulation*.

[4] Pretre R., Turina M. I. (2000). Valve disease: cardiac valve surgery in the octogenarian. *Heart*.

[5] Cheitlin M. D., Gerstenblith G., Hazzard W. R. (2001). Do existing databases answer clinical questions about geriatric cardiovascular disease and stroke?. *The American Journal of Geriatric Cardiology*.

[6] Aziz S., Grover F. L. (1999). Cardiovascular surgery in the elderly. *Cardiology Clinics*.

[7] Khan J. H., McElhinney D. B., Hall T. S., Merrick S. H. (1998). Cardiac valve surgery in octogenarians. *Archives of Surgery*.

[8] International Diabetes Federation (2019). *IDF Diabetes Atlas*.

[9] Selig J. I., Ouwens D. M., Raschke S. (2019). Impact of hyperinsulinemia and hyperglycemia on valvular interstitial cells—a link between aortic heart valve degeneration and type 2 diabetes. *Biochimica et Biophysica Acta—Molecular Basis of Disease*.

[10] World Health Organization (2006). *Definition and Diagnosis of Diabetes Mellitus and Intermediate and Hyperglycaemia. Report of a WHO/IDF Consultation*.

[11] Lang R. M., Bierig M., Devereux R. B. (2005). Recommendations for chamber quantification: a report from the American Society of Echocardiography’s guidelines and standards committee and the chamber quantification writing group, developed in conjunction with the European Association of Echocardiography, a branch of the European Society of Cardiology. *Journal of the American Society of Echocardiography*.

[12] Baumgartner H., Hung J., Bermejo J. (2009). Echocardiographic assessment of valve stenosis: EAE/ASE recommendations for clinical practice. *European Journal of Echocardiography*.

[13] Zoghbi W., Enriquez-Sarano M., Foster E. (2003). Recommendations for evaluation of the severity of native valvular regurgitation with two-dimensional and Doppler echocardiography. *Journal of the American Society of Echocardiography*.

[14] Kwan J., Shiota T., Agler D. A. (2003). Geometric differences of the mitral apparatus between ischemic and dilated cardiomyopathy with significant mitral regurgitation: real-time three-dimensional echocardiography study. *Circulation*.

[15] Grigioni F., Enriquez-Sarano M., Zehr K. J., Bailey K. R., Tajik A. J. (2001). Ischemic mitral regurgitation: long-term outcome and prognostic implications with quantitative Doppler assessment. *Circulation*.

[16] Li J. S., Sexton D. J., Mick N. (2000). Proposed modifications to the Duke criteria for the diagnosis of infective endocarditis. *Clinical Infectious Diseases*.

[17] Akram M. R., Chan T., McAuliffe S., Chenzbraun A. (2009). Non-rheumatic annular mitral stenosis: prevalence and characteristics. *European Journal of Echocardiography*.

[18] Marijon E., Ou P., Celermajer D. S. (2007). Prevalence of rheumatic heart disease detected by echocardiographic screening. *New England Journal of Medicine*.

[19] Reis G., Motta M. S., Barbosa M. M., Esteves W. A., Souza S. F., Bocchi E. A. (2004). Dobutamine stress echocardiography for noninvasive assessment and risk stratification of patients with rheumatic mitral stenosis. *Journal of the American College of Cardiology*.

[20] Warnes C. A., Williams R. G., Bashore T. M. (2008). ACC/AHA 2008 guidelines for the management of adults with congenital heart disease: executive summary. *Circulation*.

[21] Deedwania P. C. (2005). Diabetes and hypertension, the deadly duet: importance, therapeutic strategy, and selection of drug therapy. *Cardiology Clinics*.

[22] Collart F., Feier H., Kerbaul F. (2005). Valvular surgery in octogenarians: operative risks factors, evaluation of euroscore and long term results. *European Journal of Cardio-Thoracic Surgery*.

[23] Bose A. K., Aitchison J. D., Dark J. H. (2007). Aortic valve replacement in octogenarians. *Journal of Cardiothoracic Surgery*.

[24] Bakaeen F. G., Chu D., Huh J., Carabello B. A. (2010). Is an age of 80 years or greater an important predictor of short-term outcomes of isolated aortic valve replacement in veterans?. *The Annals of Thoracic Surgery*.

[25] Florath I., Albert A., Boening A., Ennker I. C., Ennker J. (2010). Aortic valve replacement in octogenarians: identification of high-risk patients. *European Journal of Cardio-Thoracic Surgery*.

[26] Mathew J. P., Fontes M. L., Tudor I. C. (2004). A multicenter risk index for atrial fibrillation after cardiac surgery. *Journal of the American Medical Association*.

[27] Filardo G., Hamilton C., Hamman B., Hebeler R. F., Adams J., Grayburn P. (2010). New-onset postoperative atrial fibrillation and long-term survival after aortic valve replacement surgery. *The Annals of Thoracic Surgery*.

[28] Coylewright M., Palmer R., O’Neill E. S., Robb J. F., Fried T. R. (2016). Patient-defined goals for the treatment of severe aortic stenosis: a qualitative analysis. *Health Expectations*.

[29] Green P., Rosner G. F., Schwartz A. (2013). Valvular heart disease in older adults: evolving technology to meet the needs of aging patients. *Aging Health*.

[30] Song F., Liu F. Z., Liang Y. F. (2019). Clinical, sonographic characteristics and long-term prognosis of valvular heart disease in elderly patients. *Journal of Geriatric Cardiology*.

[31] Esteves A. F., Brito D., Rigueira J. (2018). Profiles of hospitalized patients with valvular heart disease: experience of a tertiary center. *Revista Portuguesa de Cardiologia*.

[32] Lindman B. R., Otto C. M. (2013). Time to treat hypertension in patients with aortic stenosis. *Circulation*.

[33] Sarwar N., Sarwar N., Gao P. (2010). Diabetes mellitus, fasting blood glucose concentration, and risk of vascular disease: a collaborative meta-analysis of 102 prospective studies. *Lancet (London, England)*.

[34] Luitse M. J., Biessels G. J., Rutten G. E., Kappelle L. J. (2012). Diabetes, hyperglycaemia, and acute ischaemic stroke. *The Lancet Neurology*.

[35] van Sloten T. T., Sedaghat S., Carnethon M. R., Launer L. J., Stehouwer C. D. A. (2020). Cerebral microvascular complications of type 2 diabetes: stroke, cognitive dysfunction, and depression. *The Lancet Diabetes & Endocrinology*.

[36] Wardlaw J. M., Smith C., Dichgans M. (2019). Small vessel disease: mechanisms and clinical implications. *The Lancet Neurology*.

[37] Cosentino F., Grant P. J., Aboyans V. (2020). 2019 ESC guidelines on diabetes, pre-diabetes, and cardiovascular diseases developed in collaboration with the EASD. *European Heart Journal*.

[38] Nichols G. A., Hillier T. A., Erbey J. R., Brown J. B. (2001). Congestive heart failure in type 2 diabetes: prevalence, incidence, and risk factors. *Diabetes Care*.

[39] Seferovic P. M., Paulus W. J. (2015). Clinical diabetic cardiomyopathy: a two-faced disease with restrictive and dilated phenotypes. *European Heart Journal*.

[40] Kitada M., Zhang Z., Mima A., King G. L. (2010). Molecular mechanisms of diabetic vascular complications. *Journal of Diabetes Investigation*.

[41] Reardon M. J., Van Mieghem N. M., Popma J. J. (2017). Surgical or transcatheter aortic-valve replacement in intermediate-risk patients. *New England Journal of Medicine*.

[42] Bucerius J., Gummert J. F., Walther T. (2003). Impact of diabetes mellitus on cardiac surgery outcome. *The Thoracic and Cardiovascular Surgeon*.

[43] Halkos M. E., Kilgo P., Lattouf O. M. (2010). The effect of diabetes mellitus on in-hospital and long-term outcomes after heart valve operations. *The Annals of Thoracic Surgery*.

[44] Biniecka M., Canavan M., McGarry T. (2016). Dysregulated bioenergetics: a key regulator of joint inflammation. *Annals of the Rheumatic Diseases*.

[45] Lumeng C. N., Deyoung S. M., Saltiel A. R. (2007). Macrophages block insulin action in adipocytes by altering expression of signaling and glucose transport proteins. *American Journal of Physiology-Endocrinology and Metabolism*.

[46] Saisho Y. (2015). Metformin and inflammation: its potential beyond glucose-lowering effect. *Endocrine, Metabolic & Immune Disorders—Drug Targets*.

[47] Ormseth M. J., Oeser A. M., Cunningham A. (2013). Peroxisome proliferator-activated receptor *γ* agonist effect on rheumatoid arthritis: a randomized controlled trial. *Arthritis Research and Therapy*.

[48] Kim S. C., Schneeweiss S., Glynn R. J., Doherty M., Goldfine A. B., Solomon D. H. (2015). Dipeptidyl peptidase-4 inhibitors in type 2 diabetes may reduce the risk of autoimmune diseases: a population-based cohort study. *Annals of the Rheumatic Diseases*.

