# Supplementary material for: Clinical Characteristics and Long-Term Prognosis of Elderly Valvular Heart Disease Patients with Diabetes Mellitus: Five-Year Experience from a Single-Center Study of Southern China
Source: Cardiol Res Pract. 2021 Oct 27;2021:2558639. doi: 10.1155/2021/2558639 (PMC8566085; doi:10.1155/2021/2558639)
Supplement: Supplementary Materials — Table S1: multivariable cox regression analyses for all-cause mortality in elderly VHD patients. Table S2: multivariable cox regression analyses for ischemic stroke in elderly VHD patients. Table S3: multivariable cox regression analyses for heart failure rehospitalization in elderly VHD patients. [file 2558639.f1.docx]

Table S1. Multivariable Cox Regression Analyses for All-Cause Mortality in Elderly VHD Patients.

|  | aHR | 95% CI | *p* value |
| --- | --- | --- | --- |
| Age | 1.04 | 1.00-1.09 | 0.032 |
| COPD | 1.33 | 0.78-2.28 | 0.292 |
| Rheumatic VHD | 1.36 | 0.74-2.50 | 0.317 |
| Hypertension | 1.77 | 0.98-3.19 | 0.059 |
| Atrial fibrillation | 1.33 | 0.76-2.30 | 0.317 |
| NYHA class | 1.62 | 1.10-2.39 | 0.014 |
| LAd | 1.02 | 0.99-1.05 | 0.143 |
| Aortic regurgitation | 1.82 | 0.97-3.41 | 0.063 |
| Beta blocker | 1.46 | 0.64-3.30 | 0.367 |
| Diuretic | 1.46 | 0.87-2.45 | 0.152 |

Note. COPD indicates chronic obstructive pulmonary disease; VHD, valvular heart disease; NYHA, New York Heart Association; LAd, left atrial diameter; aHR, adjusted hazard ratio; CI, confidence interval.

Table S2 Multivariable Cox Regression Analyses for Ischemic Stroke in Elderly VHD Patients.

|  | aHR | 95% CI | *p* value |
| --- | --- | --- | --- |
| Age | 1.04 | 1.01-1.07 | 0.017 |
| Hypertension | 1.84 | 1.08-3.15 | 0.025 |
| Atrial fibrillation | 2.49 | 1.61-3.87 | ＜0.001 |
| LVEF | 1.01 | 0.99-1.03 | 0.341 |
| LVDd | 0.98 | 0.95-1.01 | 0.123 |
| Aortic stenosis | 0.46 | 0.22-0.96 | 0.039 |
| Diabetes mellitus | 1.74 | 1.14-2.66 | 0.010 |

Note. VHD indicates valvular heart disease; LVEF, left ventricular ejection fraction; LVDd, left ventricular internal diameter at end-diastole; aHR, adjusted hazard ratio; CI, confidence interval.

Table S3 Multivariable Cox Regression Analyses for Heart Failure Rehospitalization in Elderly VHD Patients.

|  | aHR | 95% CI | p value |
| --- | --- | --- | --- |
| Infective VHD | 3.286 | 1.14-9.49 | 0.028 |
| Hypertension | 1.265 | 0.84-1.90 | 0.253 |
| Atrial fibrillation | 1.206 | 0.82-1.78 | 0.345 |
| COPD | 1.809 | 1.25-2.63 | 0.002 |
| Chronic kidney disease | 1.169 | 0.75-1.83 | 0.496 |
| LVDd | 1.000 | 0.98-1.02 | 0.731 |
| NYHA | 1.536 | 1.18-2.00 | 0.001 |
| Aortic regurgitation | 1.580 | 1.04-2.41 | 0.034 |
| Diuretic | 1.109 | 0.74-1.67 | 0.618 |
| LAd | 1.010 | 0.99-1.04 | 0.173 |
| Diabetes mellitus | 1.920 | 1.34-2.74 | ＜0.001 |

Note. VHD indicates valvular heart disease; COPD, chronic obstructive pulmonary disease; LVDd, left ventricular internal diameter at end-diastole; NYHA, New York Heart Association; LAd, left atrial diameter; aHR, adjusted hazard ratio; CI, confidence interval.
